# The oral microbiome of early stage Parkinson’s disease and its relationship with functional measures of motor and non-motor function

**DOI:** 10.1371/journal.pone.0218252

**Published:** 2019-06-27

**Authors:** Dragos Mihaila, Jordan Donegan, Sarah Barns, Daria LaRocca, Qian Du, Danny Zheng, Michael Vidal, Christopher Neville, Richard Uhlig, Frank A. Middleton

**Affiliations:** 1 Department of Neurology, SUNY Upstate Medical University, Syracuse, New York, United States of America; 2 Department of Neuroscience & Physiology, SUNY Upstate Medical University, Syracuse, New York, United States of America; 3 Quadrant Biosciences, Inc., Syracuse, New York, United States of America; 4 Department of Department of Physical Therapy Education, SUNY Upstate Medical University, Syracuse, New York, United States of America; 5 Department of Psychiatry & Behavioral Sciences, SUNY Upstate Medical University, Syracuse, New York, United States of America; 6 Department of Biochemistry & Molecular Biology, SUNY Upstate Medical University, Syracuse, New York, United States of America; 7 Department of Pediatrics, SUNY Upstate Medical University, Syracuse, New York, United States of America; University of Illinois at Urbana-Champaign, UNITED STATES

## Abstract

Changes in the function and microbiome of the upper and lower gastrointestinal tract have been documented in Parkinson’s disease (PD), although most studies have examined merely fecal microbiome profiles and patients with advanced disease states. In the present study we sought to identify sensitive and specific biomarkers of changes in the oral microbiome of early stage PD through shotgun metatranscriptomic profiling. We recruited 48 PD subjects and 36 age- and gender-matched healthy controls. Subjects completed detailed assessments of motor, cognitive, balance, autonomic and chemosensory (smell and taste) functions to determine their disease stage. We also obtained a saliva sample for profiling of microbial RNA and host mRNA using next generation sequencing. We found no differences in overall alpha and beta diversity between subject groups. However, changes in specific microbial taxa were observed, including primarily bacteria, but also yeast and phage. Nearly half of our findings were consistent with prior studies in the field obtained through profiling of fecal samples, with others representing highly novel candidates for detection of early stage PD. Testing of the diagnostic utility of the microbiome data revealed potentially robust performance with as few as 11 taxonomic features achieving a cross-validated area under the ROC curve of 0.90 and overall accuracy of 84.5%. Bioinformatic analysis of 167 different metabolic pathways supported shifts in a small set of distinct pathways involved in amino acid and energy metabolism among the organisms comprising the oral microbiome. In parallel with the microbial analysis, we also examined the evidence for changes in human salivary mRNAs in the same subjects. This revealed significant changes in a set of 9 host mRNAs, several of which mapped to various brain functions and showed correlations with some of the significantly changed microbial taxa. Unexpectedly, we also observed robust correlations between many of the microbiota and functional measures, including those reflecting cognition, balance, and disease duration. These results suggest that the oral microbiome may represent a highly-accessible and informative microenvironment that offers new insights in the pathophysiology of early stage PD.

## Introduction

The etiology of Parkinson’s Disease (PD) is complex. Many factors, including genes, lifestyle, age, sex and even epigenetic factors are all known to affect the risk of PD and its progression [1–3]. Historically, most studies on the etiology of PD have focused on factors that influence midbrain dopaminergic neurons and their projections to the striatum (reviewed in [4]). Over the past decade, however, it has become increasingly clear that disturbances and pathology within the upper and lower gastrointestinal (GI) system in PD actually precede the pathology in the central nervous system (CNS)[1, 4–6]. Specifically, according to Braak and colleagues [5, 7] the submandibular salivary gland and lower esophagus appear to have a high frequency of alpha-synuclein associated Lewy Body (LB) pathology, followed by the stomach, small intestine and colon. Moreover, submandibular biopsy specimens from living PD subjects have also been recently reported to contain LBs [8, 9]. Notably, the appearance of LBs in these sites is thought to coincide with symptoms of GI dysfunction before the onset of motor symptoms in a large proportion of PD subjects, a feature that has also been seen in mouse and non-human primate models of PD [10].

The CNS communicates with the GI tract through the gut-brain axis. This axis includes not only neural, but also immunologic and endocrine connections. Within the upper and lower GI tract, the enteric nervous system (ENS) shares many of the same neurotransmitters as the CNS, including dopamine and serotonin [11], which are synthesized by specific populations of ENS neurons and play roles in GI motility. Notably, the ENS also contains extensive populations of glial cells, which also influence GI motility, and play diverse roles in inter-cellular communication, inflammatory processes, and responses to infection or changes in microbial composition [12].

Current theories studies suggest that once the initial LB pathology has been established in the GI tract, it can spread to the CNS in a prion-like manner along vagal nerve fibers [4][13]. The major component of LBs is alpha-synuclein. Normally, alpha-synuclein is thought to play an important role in modulating neurotransmitter release through interactions with the pre-synaptic SNARE complex [14]. However, marked increases in alpha synuclein expression, mutations in the sequence of alpha synuclein, or even simply high levels of oxidative stress in the local microenvironment, all seem capable of promoting formation of pathological aggregates by interfering with normal turnover of alpha-synuclein via the ubiquitin-proteasome system [4, 15–17].

The microenvironment of the GI tract is strongly affected by the local microbiome. While there is a wide range of diversity in the microbiota inhabiting different levels of the GI tract, the microbes within each level normally play an important commensal role in modulating health. Specifically, they regulate immune function, modulate gut-brain interactions, synthesize essential enzymatic cofactors and metabolic intermediates, and help prevent colonization by pathogenic bacteria. In subjects with PD, however, disturbances in the microbial composition of the GI tract have now been implicated in hastening the progression of the disease by contributing to LB formation (reviewed in [18, 19]. One theory posits that PD patients have increased gut permeability compared to healthy controls, and this is associated with increased bacterial translocation out of the intestinal lumen, where their cell membranes can release lipopolysaccharide (LPS) and other byproducts that stimulate robust inflammatory responses [20]. While it is unclear if the shifts in microbial content are a direct cause or consequence of the increased inflammation and impaired GI barrier function in PD, it does appear that a proinflammatory state in the GI tract is associated with enhanced spread of LB pathology [18].

Given its potential importance in understanding the complete pathophysiology of PD, it is not surprising that much attention has recently focused on defining the microbiome changes in PD. However, the vast majority of studies published on this topic have examined only lower GI or fecal samples, and examined these in subjects with well-established motor symptoms or advanced disease states. The purpose of the present study was to comprehensively examine the oral microbiome in subjects with early stage PD in comparison with healthy age-matched controls, and attempt to relate the levels of specific microbiota to specific clinical and demographic features. Because LB pathology has been shown to occur in the oropharyngeal cavity and because many components of the upper GI tract are under the control of dopaminergic or noradrenergic inputs which are also affected in PD, we hypothesized that subjects with PD may have altered oropharyngeal status in terms of the control of swallowing, speech or salivation, and that this may alter the salivary microbiome in a reproducible manner that differentiates them from control subjects. Such differences might reflect changes in salivary pH or secretion of salivary enzymes and proteins that directly alter the oral microbiota. We also reasoned that there might be evidence for either causal or compensatory changes in the host to such microbial changes, which could be assessed by measurement of human oral mRNA.

## Materials and methods

### Study design

This was a cross-sectional case-control design employing high throughput RNA sequencing to examine salivary microbial RNAs in subjects with early stage Parkinson’s disease and healthy age and gender matched controls.

### Subject ascertainment

This study was approved by the Institutional Review Board for the Protection of Human Subjects (IRB) at SUNY Upstate Medical University in Syracuse, NY. Informed written consent was obtained for all human subjects. Subjects were recruited from the greater Syracuse and Upstate New York area and received copies of the study description, consent documentation, and a comprehensive health and symptom questionnaire packet prior to their study visit. The questionnaire packet encompassed a detailed medical and health history and six standardized instruments: (1) The Movement Disorder Society–Unified Parkinson’s Disease Rating Scale, Part I (MDS-UPDRS-I)[21], referred to as Non-Motor Aspects of Experiences of Daily Living; (2) The MDS-UPDRS, Part II, referred to as the Motor Experiences of Daily Living [21]; (3) The Scales for Outcomes in Parkinson’s Disease Autonomic Questionnaire (SCOPA-AUT)[22]; (4) The Parkinson’s Disease Quality of Life Scale (PDQUALIF)[23]; (5) The Non-Motor Symptom Questionnaire (NMS)[24]; and (6) The Beck Depression Inventory (BDI)[25].

#### Inclusion/exclusion criteria

None of the participants had active dental caries, periodontal disease, or diseases of the nasopharyngeal and oropharyngeal cavity within the past 2 weeks or antibiotic use in the past month prior to sample collection. Subjects included in the Parkinson’s disease (PD) group had been previously diagnosed by a neurologist and met the general diagnostic criteria for late-onset PD, including bradykinesia, and rigidity or a resting tremor [21]. Exclusion criteria included a history of neuroleptic use or moderate to severe traumatic brain injury (TBI) that might have contributed to trauma-induced parkinsonism. Because our focus was on early stage PD, we also excluded subjects with a Hoehn & Yahr staging score of 4 or more. Control subjects were included if they had no prior history of major medical procedures or conditions, were never on PD medications or suspected of having a movement disorder, and did not have any first-degree relatives with PD.

#### Functional evaluation

All PD subjects were evaluated using the Motor Examination (Part III) of the MDS-UPDRS by a movement disorder specialist or trained Ph.D.-level evaluator. Permission to use the UPDRS was obtained from the Movement Disorder Society. PD subjects also completed a spiral tracing test and cursive handwriting test to screen for persistent non-resting tremor as well as micrographia, and underwent resting tremor measurements in both hands while wearing a highly sensitive accelerometer (sampling frequency = 250 Hz). Height, weight, blood pressure and pulse were obtained on all subjects. All subjects then completed a detailed sensory, motor, cognitive, and balance assessment that assessed several functions shown to have potential diagnostic or screening utility [26–31]: (1) 12-item Modified Brief Smell Identification Test (mBSIT, Sensonics, Inc.); (2) a 10-item taste test (for sweet, salty, sour and bitter solutions at threshold and 3x threshold concentrations [32]); (3) Trailmaking A test; (4) Trailmaking B test; (5) Digit Span Forward test; (6) Digit Span Reverse test; (7) Simple Reaction Time (SRT); (8) Procedural Reaction Time test (PRT); (9) Go/No-Go test (GNG); and (10) balance/body sway measurements (30 seconds duration) with their shoes off in 10 different postures, while wearing an inertial sensor/accelerometer around their waste. With the exception of the two sensory measures, these items were part of ClearEdge, an integrated tablet-based FDA-listed functional assessment system (Quadrant Biosciences, Inc.) that incorporates three simple and complex reaction time measures (SRT, PRT, GNG) from the DANA BrainVitals battery (AnthoTronix, Inc.) along with the measurements of postural sway and cognitive performance [33, 34]. The postures that were used were as follows: Two legs side by side, eyes open, on a hard surface (TLEO); Two legs side by side, eyes closed, on a hard surface (TLEC); Tandem stance, eyes open, on a hard surface (TSEO); Tandem stance, eyes closed, on a hard surface (TSEC); Two legs side by side, eyes open, on a foam pad (TLEOFP); Two legs side by side, eyes closed, on a foam pad (TLECFP); Tandem stance, eyes open, on a foam pad (TSEOFP); Tandem stance, eyes closed, on a foam pad (TSECFP); a simple dual task involving tandem stance, eyes open, on a hard surface while holding the tablet device (TSEOHT); and a complex dual task involving completion of Trailmaking B while holding the tablet, with two legs side by side, eyes open, on a hard surface (TLEOCT).

Raw demographic data were collected for all subjects. The functional Balance, Motor, and Cognitive score data were converted to z scores by direct comparison of each subject to a pooled reference value that represented the trimmed mean of the control group after removal of any outlier data points (data points exceeding ± 2 standard deviations from the mean of the control group). To be more conservative, however, these outlier points were retained for all between group comparisons. The resulting set of 35 demographic and functional variables were then screened separately for normality in PD and controls using the Shapiro-Wilk Test, with a criterion set at 0.05. This indicated that more than half of the variables failed the normality test in both subject groups. Accordingly, we used a non-parametric Mann-Whitney test to compare the group median ranks on all demographic and functional scores, with a false discovery rate (FDR) set at q<0.1 to control for multiple testing. For simplicity, all demographic and functional differences reported are either mean percentage or z score differences between the groups. Additional clinical and functional data was also obtained on the PD subjects and compiled as relative frequencies or raw values and instrument scores. These values were cross-referenced where appropriate to established cutoff values for mild to moderate PD symptom severity based on published literature.

#### Saliva collection and processing

**Fig 1:**
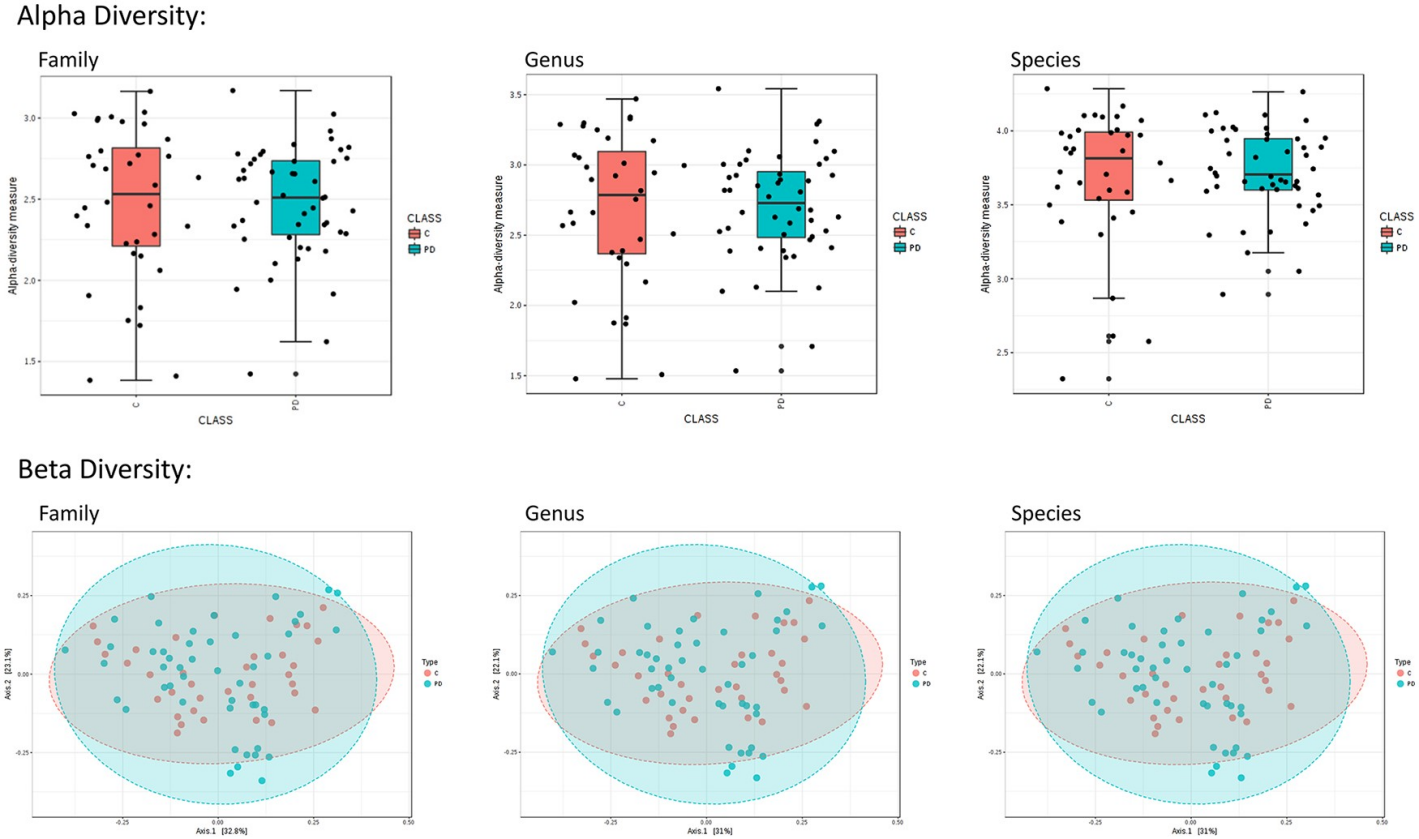
No differences in family, genus or species biodiversity measures in early stage PD. Whisker box plots indicate mean and range of Shannon alpha diversity (upper) and Bray-Curtis dissimilarity measures (lower) for the family, genus, and species levels of classification.

**Fig 2:**
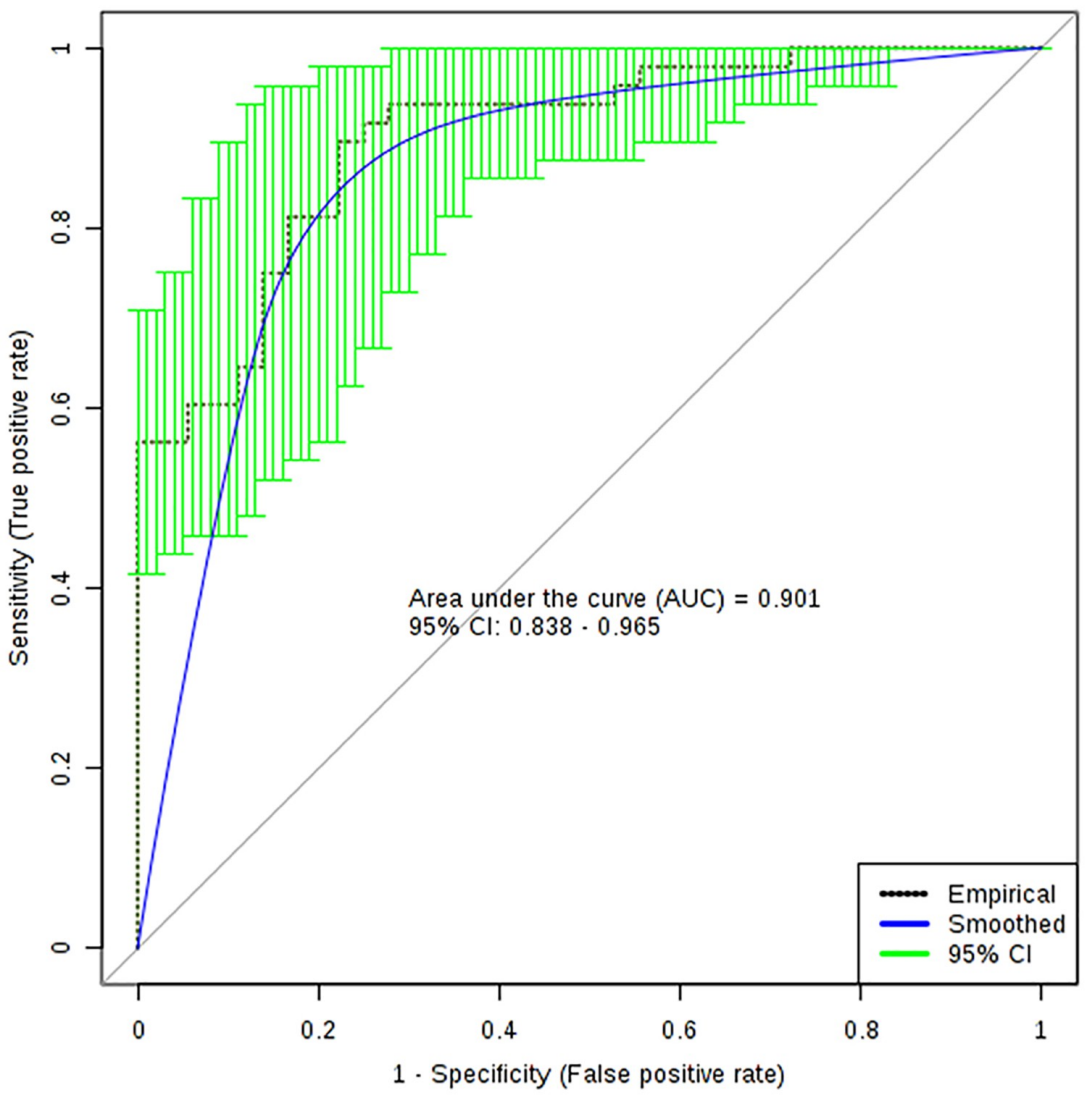
ROC curve performance using the oral microbiome. Empirical ROC performance during cross-validation and its 95^th^ percentile confidence interval are shown. Overall accuracy was 84.5%.

**Fig 3:**
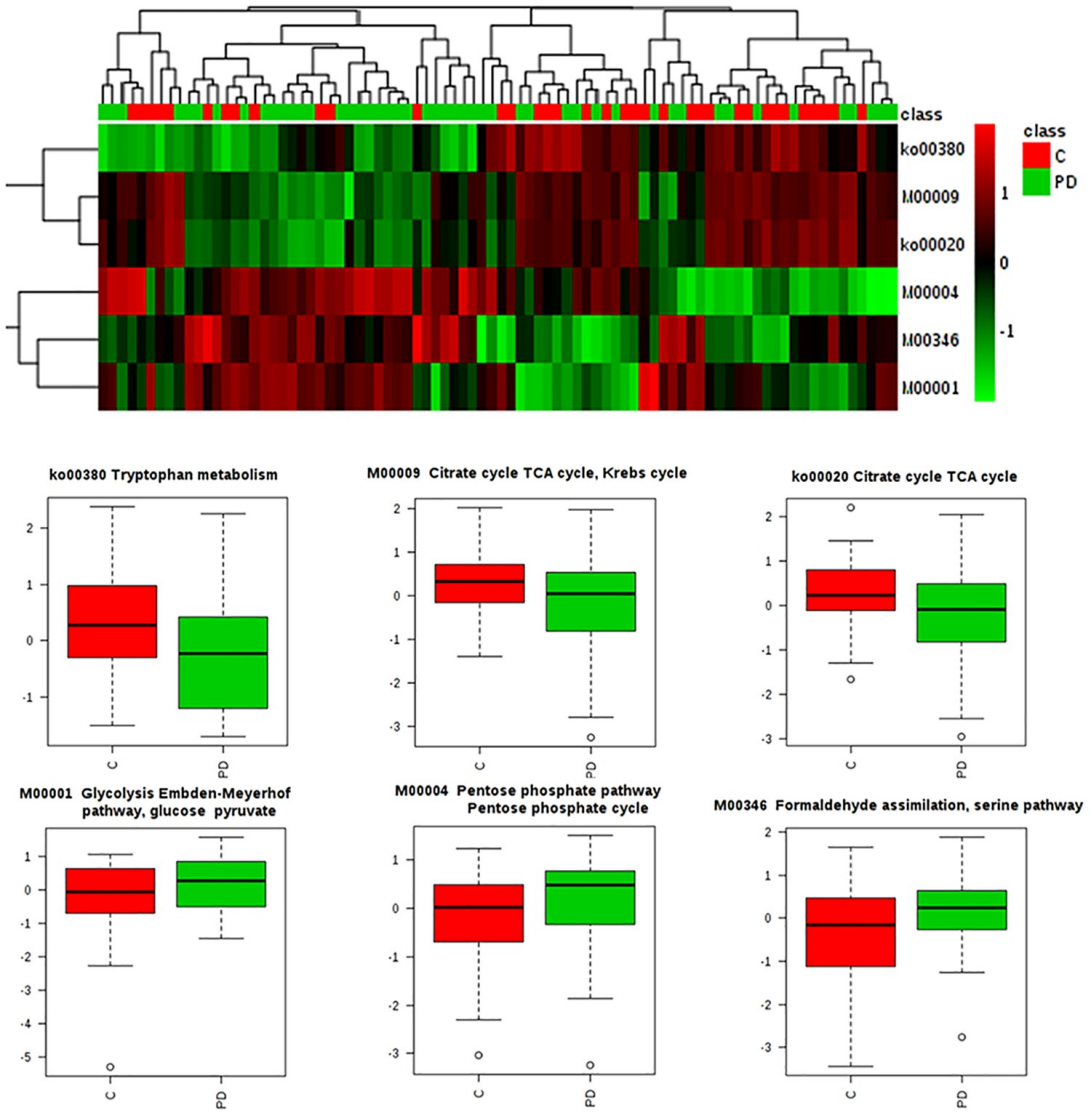
Metabolic pathway changes in oral microbiome of early stage PD. Only 6 of the 167 KEGG pathways were changed when examining transcripts from the oral microbiome in PD subjects compared with controls.

Subjects provided a saliva sample by expectoration into an OraGene RNA (RE-100) collection vial (DNA Genotek, Ottawa, ON). At least 30 minutes had elapsed between the time of last food or drink consumption and saliva collection. Before collecting saliva samples, each subject rinsed their mouth with bottled water. Approximately 1 mL of saliva was obtained from each participant. Samples were stored at room temperature during the study visit and then at 4C until processing. A Trizol method was used to purify the salivary RNA and a second round of purification was followed using an RNEasy mini column (Qiagen). Yield and quality of the RNA samples was assessed with the RNA NanoChip on the Agilent Bioanalyzer prior to library construction using the Illumina TruSeq Small RNA Sample Prep protocol (Illumina; San Diego, California). Identification and quantification of microbial RNA was performed using next generation sequencing (NGS) on a NextSeq 500 instrument (Illumina). Sets of 48 samples were indexed together at a targeted depth of 10 million single-end 50 bp reads per sample. De-indexing, adapter trimming and quality control metrics were obtained from Partek Flow software. Alignment of microbial transcripts was performed using the k-SLAM software[35], which references the NCBI Taxonomy database, after filtering to remove miRNAs and other RNAs that aligned to the human transcriptome. Taxa were defined by their family, genus, species, and subspecies (when available). All of the raw taxa abundance measurements are provided as supplemental data (S1 Table). Moreover, unlike pure 16S-based sequencing, k-SLAM software does not merely rely on a single gene to define taxa following shotgun sequencing. Instead, it utilizes genome-wide data and performs optimal alignments using k-mer based approaches. Accordingly, we also include a list of the raw read alignments for the identified transcripts, according to their KEGG Orthology (KO) identifiers, as supplemental data (S2 Table). Finally, to directly investigate the potential for host mRNA interactions with the microbiome, we aligned the NGS data to the human transcriptome (hg38), using the Shrimp2 aligner in Partek Flow. The raw alignment counts for all mRNAs is reported as supplemental data (S3 Table). The microbial RNA present in raw counts of 10 or more in at least 10% of samples were interrogated for differences between subject groups in overall richness using the Shannon alpha diversity and Bray-Curtiss beta diversity metrics (Fig 1). A phylogenetic composition analysis was performed prior to mining for group differences using a dendrogram with a Spearman distance metric (S1 Fig) The set of genus and species data were then examined for between group differences using the Mann-Whitney test with false discovery correction (FDR<0.05) and for the ability to completely distinguish the subjects in a binomial classification test using logistic regression with receiver operating characteristic (ROC) curve analysis (with 10-fold cross-validation) (Fig 2). The separability of the samples according to diagnosis group was further examined using a Partial Least Squares Discriminant Analysis (PLSDA) (S2 Fig). The biological significance of differential microbial transcript abundance was assessed using KEGG Pathway mapping as well as hierarchical clustering analysis within MicrobiomeAnalyst and MetaboAnalyst R packages[36, 37] (Fig 3). Summary tables of the 40 most abundant overall microbial RNAs as well as the 40 most differential microbial RNAs (as identified by their KO ID) are provided as supplemental data (S4 and S5 Tables).

To investigate potential differences in host mRNA abundance in PD subjects, we first filtered the raw data to include only human mRNAs present with at least 10 raw reads in at least 10% of the samples. Then, genes present above the median raw read count across samples were sum normalized, log2 scaled and z-score transformed. A volcano plot was used to investigate differences between groups for approximately 3,800 genes using a Student’s t test with FDR set at q < 0.20 (S3 Fig). The differentially expressed human mRNAs (S6 Table) were examined across samples and groups using hierarchical clustering with a Pearson distance metric (S3 Fig). Significantly changed human genes were examined for interactions and enrichment in biological ontologies using the STRING database. Results were visualized in a gene-gene interaction network (S3 Fig), and enriched Gene Ontologies listed in table form (S7 Table). To explore potential associations between the changed microbial taxa and human mRNAs, we performed Spearman rank-based correlations and report FDR-corrected P values (S8 Table). Correlations between different microbial and functional and demographic measures were assessed in an exploratory manner by Pearson product-moment correlation analysis (S9 Table).

## Results

### Participants

A total of 84 subjects completed the study, including 36 healthy controls with no history of movement disorder and 48 subjects with early stage PD (Table 1).

**Table 1:** Subject demographic and clinical measures.

Group	% Male	Age	Height	Weight	BMI	Systolic BP	Pulse	Ave Sleep
Parkinson (n = 48)	60.4	69.5 yrs	67.1”	174.9 lbs	26.9	131.4	*72.2	7.0 hrs
Control (n = 36)	55.6	68.5 yrs	67.1”	168.7 lbs	26.2	130.3	66.6	7.5 hrs

* Significant (FDR < 0.04) difference versus Control group

### Functional outcomes

Among the PD subjects, the average duration of a diagnosis was 3.4 years (SE ± 0.56 years), with an average Hoehn & Yahr Stage of 1.92, and average scores for subscales of the MDS-UPDRS, NMS, SCOPA-AUT, PDQUALIF, and BDI all falling in established ‘mild’ ranges for those instruments according to published criteria (UPDRS-I 10.0, UPDRS-II 8.6, UPDRS-III 23.9) Most PD subjects (69%) were observed to have resting tremor and 87% were on PD medication (Table 2). Notably, more than 95% of our PD subjects had evidence of upper or lower GI disturbance (Table 2).

**Table 2:** PD subject characteristics.

Scale/Subscale	Average	Mild/Moderate Cutoff Mild/Moderate	Reference
UPDRS-I	10.0	10–11	[38]
UPDRS-II	8.6	12–13	[38]
UPDRS-III	23.9	32–33	[38]
Hoehn & Yahr Stage	1.92	3.0	[39]
Duration of illness	3.44		
Noted Resting Tremor %	68.8		
Anti-PD medication %	87.5		
Sleep Dysfunction %	83.3		
Oropharyngeal Dysfunction %	85.4		
Thermoregulatory, Vasomotor Dysfunction %	90.0		
GI or Urinary Dysfunction %	95.8		
NMS Questionnaire	8.0	8.8–12.0	[40]
SCOPA-AUT	12.0	16–17	[22]
PDQUALIF	35.25	37.7–38.8	[23]
Beck Depression Inventory	7.4	13	[25]

Compared with healthy control subjects, the PD subjects in our cohort were found to exhibit significant changes in several indices of motor, cognitive and sensory function. Specifically, the early stage PD subjects showed significant increases in completion times for the Trailmaking A and B tasks, and corresponding significant decreases in Trailmaking A and B completion scores (Table 3). These deficits were present in the absence of a significant change in Simple Reaction Time Score, although a trend for slower reaction times was apparent. Complementing these findings, the Procedural Reaction Time (PRT) Score was also significantly decreased in the DANA Brain Vitals set of measures (SRT, PRT, GNG) (Table 3).

**Table 3:** Motor, cognitive, and sensory outcome measures.

Measure	PD	CTRL	z score diff (or % diff)	FDR
Trailmaking A (Completion Time)	1.47	0.20	1.27	**0.0034**
Trailmaking A (Completion Score)	-0.41	0.0	-0.41	**0.0768**
Trailmaking B (Completion Time)	1.84	0.36	1.48	**0.0653**
Trailmaking B (Completion Score)	-0.88	0.0	-0.88	**0.0024**
Digit Span Forward (Score)	-0.15	0.0	-0.15	0.4513
Digit Span Reverse (Score)	-0.08	0.0	-0.08	04590
Two Legs EO (TLEO Balance Score)	-1.80	0.0	-1.80	**0.0026**
Two Legs EC (TLEC Balance Score)	-0.39	0.0	-0.39	**0.0250**
Tandem Stance EO (TSEO Balance Score)	-0.87	0.0	-0.87	**0.0258**
Tandem Stance EC (TSEC Balance Score)	-0.30	-0.25	-0.05	0.4724
Two Legs EO Foam Pad (TLEOFP Balance Score)	-0.38	0.0	-0.38	**0.0665**
Two Legs EC Foam Pad (TLECFP Balance Score)	-0.63	-0.22	-0.41	0.1468
Tandem Stance EO Foam Pad (TSEOFP Balance Score)	-0.26	-0.27	0.01	0.4824
Tandem Stance EC Foam Pad (TSECFP Balance Score)	0.55	0.0	0.55	**0.0245**
Holding Tablet Dual Task (TSEOHT Balance Score)	-0.24	0.0	-0.24	0.4196
Trailmaking B_Dual Task (Balance Score)	-1.50	-1.08	-0.43	0.1578
Trailmaking B_Dual Task (Completion Score)	-0.42	0.0	-0.42	**0.0463**
Trailmaking B Dual Task (Completion Time)	1.65	0.34	1.31	**0.0266**
Simple Reaction Time (SRT Score)	-0.60	-0.12	-0.48	**0.0622**
Procedural Reaction Time (PRT Score)	-0.96	-0.10	-0.85	**0.0082**
Go/NoGo (GNG Score)	-0.65	-0.11	-0.54	**0.0265**
Taste test (Raw Score/10)	6.81	8.2	-17%	**0.0000**
Smell test (Raw Score/12)	7.42	10.3	-28%	**0.0000**

Note: Measures with FDR < 0.10 were considered significant

#### Balance scores

PD subjects were found to exhibit increased body sway (decreased score) in four of the balance measures (TLEO, TLEC, TSEO, TLEOFP) and an apparent increase in the performance of one balance measure (TSECFP) (Table 3). However, inspection of the data for this latter task, which is usually considered the most difficult, indicated that the higher scores were likely due to selection bias of more capable PD subjects, because multiple PD subjects (n = 7) were actually unable to complete it. This was also true for another task that is considered nearly as difficult (TSEOFP), where 8 PD subjects were unable to complete it and there was no overall between group difference. Thus, discounting the TSECFP and TSEOFP tasks, the overall trend when the full group scores were available was for reduced balance scores in the PD group.

#### Reaction times and cognitive scores

PD subjects demonstrated a consistent decrease in their simple and complex reaction time measures compared with Controls, as reflected in reduced performance on the SRT, PRT and GNG tasks (Table 3). Consistent with the slowed reactions times, we also observed that PD subjects took longer to complete the Trailmaking A and B tasks, and this was accompanied by reduced scores on these measures as well (Table 3). For Trailmaking B, this was also true when subjects had to perform the test in a dual task condition, while maintaining upright standing posture (Table 3).

#### Chemosensory scores

PD subjects showed highly significant decreased performance in measures of both taste and smell (Table 3). Notably, performance on these two sensory measures was also significantly correlated (Pearson’s R = 0.27; p = 0.015), although this should not be taken as evidence of a strong association between the two measures.

#### Microbial compositional analysis

Raw taxa and raw microbial RNA alignments are provided as supplemental data (S1 and S2 Tables, respectfully). Prior to comparing the groups, we examined overall phylogenetic similarity of the samples using a dendrogram analysis (S1 Fig).

#### Microbial alpha and beta diversity

After filtering to remove taxa that were less consistently observed in the saliva samples, we did not observe any significant differences in overall alpha and beta diversity (Fig 1) between the two sample groups. However, it is noteworthy to point out that the PD subjects did appear to show a slightly greater range of beta diversity values when their data were superimposed on those of control subjects (Fig 1).

#### Microbial genus and species differences

A total of 50 microbiome taxa exhibited significant differences in abundance in PD subjects compared with control subjects. These included 16 genera and 34 species, and encompassed bacteria, phage, and Eukaryotic taxa (Table 4) (FDR < 0.05). The majority of changes observed were increases in abundance (n = 36) rather than decreases in abundance (n = 14) (Table 4). Notably, 12 of the genera findings had one or more subordinate species findings, while 4 were changed in isolation (at the genus level). Among the more commonly changed bacteria species were multiple members of the Lactobacillus (n = 6) and Bifidobacterium (n = 3) genera (Table 4). We also observed a significant decrease in a single bacteriophage (Streptococcus phage PhiSpn 200), and increases in three yeast species (*Candida albicans*, *Candida dubliniensis*, and *Saccharomyces cerevisiae*) in PD subjects (Table 4).

**Table 4:** Significantly changed microbiota in early stage PD.

Taxon	Log2 Chg	Std Err	P value	FDR
**ACIDAMINOCOCCUS**	1.15	0.34	0.000765	0.023003
*Bacillus megaterium*	*-2*.*85*	*0*.*48*	*4*.*20E-09*	*7*.*66E-07*
*Bacillus sp FJAT 22090*	*1*.*69*	*0*.*38*	*1*.*01E-05*	*0*.*001226*
**BIFIDOBACTERIUM**	2.07	0.39	1.30E-07	5.08E-05
*Bifidobacterium animalis*	*1*.*31*	*0*.*41*	*0*.*001262*	*0*.*030704*
*Bifidobacterium dentium*	*1*.*94*	*0*.*49*	*7*.*23E-05*	*0*.*003984*
*Bifidobacterium longum*	*2*.*60*	*0*.*42*	*8*.*95E-10*	*3*.*22E-07*
**BRUCELLA**	1.28	0.39	0.001041	0.027124
**BUCHNERA**	-1.73	0.43	5.43E-05	0.003539
*Buchnera aphidicola*	*-1*.*79*	*0*.*45*	*7*.*85E-05*	*0*.*003984*
*Campylobacter ureolyticus*	*-1*.*52*	*0*.*40*	*0*.*000162*	*0*.*006569*
**CANDIDA**	1.90	0.43	1.29E-05	0.001263
*Candida albicans*	*1*.*86*	*0*.*47*	*8*.*09E-05*	*0*.*003984*
*Candida dubliniensis*	*1*.*52*	*0*.*39*	*9*.*28E-05*	*0*.*003984*
*Candidatus Azobacteroides*	*-1*.*33*	*0*.*36*	*0*.*000173*	*0*.*007412*
*Candidatus Azobacteroides pseudotrichonymphae*	*-1*.*29*	*0*.*37*	*0*.*000416*	*0*.*01321*
*Capnocytophaga canimorsus*	*1*.*18*	*0*.*35*	*0*.*000853*	*0*.*022227*
**CELLULOSIMICROBIUM**	1.09	0.26	1.99E-05	0.001557
*Cellulosimicrobium sp TH 20*	*1*.*12*	*0*.*26*	*1*.*36E-05*	*0*.*001417*
**CHRYSEOBACTERIUM**	-0.99	0.31	0.001328	0.032458
*Chryseobacterium sp IHB B 17019*	*-1*.*22*	*0*.*36*	*0*.*000759*	*0*.*020533*
**CLAVIBACTER**	1.18	0.30	9.74E-05	0.00504
*Clavibacter michiganensis*	*1*.*24*	*0*.*31*	*6*.*93E-05*	*0*.*003984*
*Flavobacteriaceae bacterium 3519 10*	*-1*.*21*	*0*.*36*	*0*.*000749*	*0*.*020533*
**GARDNERELLA**	1.70	0.44	0.000103	0.00504
*Gardnerella vaginalis*	*1*.*80*	*0*.*46*	*9*.*07E-05*	*0*.*003984*
*Halobacillus mangrove*	*-1*.*08*	*0*.*35*	*0*.*001858*	*0*.*04111*
**LACTOBACILLUS**	1.61	0.35	3.31E-06	0.000431
*Lactobacillus acidophilus*	*2*.*25*	*0*.*57*	*7*.*95E-05*	*0*.*003984*
*Lactobacillus fermentum*	*3*.*19*	*0*.*53*	*1*.*32E-09*	*3*.*22E-07*
*Lactobacillus plantarum*	*1*.*39*	*0*.*33*	*2*.*92E-05*	*0*.*002367*
*Lactobacillus reuteri*	*1*.*66*	*0*.*46*	*0*.*000283*	*0*.*010319*
*Lactobacillus ruminis*	*1*.*51*	*0*.*42*	*0*.*000348*	*0*.*01207*
*Lactobacillus salivarius*	*1*.*15*	*0*.*36*	*0*.*001488*	*0*.*033954*
*Lutibacter sp LPB0138*	*1*.*35*	*0*.*42*	*0*.*001143*	*0*.*028777*
**METHYLOBACTERIUM**	1.02	0.31	0.00096	0.026802
**PARASCARDOVIA**	2.09	0.42	6.11E-07	0.000119
*Parascardovia denticolens*	*2*.*16*	*0*.*43*	*5*.*76E-07*	*8*.*41E-05*
**RHODOCOCCUS**	0.72	0.23	0.001425	0.032783
*Rhodococcus sp 008*	*1*.*31*	*0*.*33*	*8*.*66E-05*	*0*.*003984*
*Saccharomyces cerevisiae*	*1*.*48*	*0*.*47*	*0*.*001479*	*0*.*033954*
**SCARDOVIA**	1.41	0.40	0.000457	0.014885
*Scardovia inopinata*	*1*.*43*	*0*.*41*	*0*.*000534*	*0*.*015583*
*Streptococcus mutans*	*1*.*43*	*0*.*41*	*0*.*000466*	*0*.*014179*
*Streptococcus sp I G2*	*-1*.*22*	*0*.*29*	*2*.*68E-05*	*0*.*002367*
*Streptococcus phage PhiSpn 200*	*-3*.*07*	*0*.*49*	*2*.*59E-10*	*1*.*89E-07*
**TORULASPORA**	1.72	0.46	0.00019	0.007412
*Torulaspora delbrueckii*	*1*.*80*	*0*.*49*	*0*.*000228*	*0*.*00877*
**WENYINGZHUANGIA**	-1.47	0.40	0.000243	0.008622
*Wenyingzhuangia fucanilytica*	*-1*.*50*	*0*.*42*	*0*.*000364*	*0*.*01207*

Significantly changed genera appear in all upper case, with significantly changes species italicized.

#### Robust classification of early stage PD based on salivary microbiota

To further probe the consistency of the group microbiome differences, we subjected the genus and species level data to logistic regression classification and area under the receiver operating characteristic curve (ROC) analysis. This indicated a strong separation of the groups was possible using a set of the microbiota data transformed into 5 ratios of two taxa plus 4 additional individual taxa (n = 11 taxa total), with an area under the curve (AUC) during training of 0.95 and an AUC during 10-fold cross-validation of 0.90 (Fig 2). Overall accuracy was 84.5% (13 misclassified subjects out of 84 total). The separability of the samples in the different diagnostic groups according to microbial taxa was further supported by the results of a PLSDA analysis, which showed nearly complete separation of PD and control subjects when viewed in a multidimensional framework (S2 Fig).

#### Changes in microbial transcription networks in early stage PD

In contrast to purely 16S-based microbiome analyses, shotgun-based RNA seq analysis identifies different taxa by genetic diversity analysis of all available RNA sequences. Examination of the 40 most abundant transcripts (identified by KO ID) indicated that more than 80% of the identified reads were from 3 rRNA genes (16S, 5S, 23S) and 3 tRNA genes (Gly, Glu, Trp) (S4 Table). Notably, only 1 of the 40 most abundant genes showed a nominally significant difference in abundance comparing PD and control subjects (K01689 Enolase, representing .03% of mapped genes, was increased 50% in PD subjects). This highlights the importance of considering the taxa from which the microbial transcripts originate, rather than the transcripts themselves. We also probed for more statistically robust differences in KO ID abundance between groups. This identified 24 KO IDs with nominally significant differences between PD and control subjects (none of which survived FDR correction), of which only 3 contained more than 0.01% of the overall reads (S5 Table). Thus, most of the potentially differential individual KO transcripts represent very low abundance microbial RNA.

In addition to probing for individual transcript alterations, we also examined possible alterations in the expression of sets of transcripts included within defined microbial metabolic pathways. A total of 167 KEGG pathways were examined using the K-Slam mapping data, of which six showed nominally significant changes (Table 5). The changes included three pathways with increased expression (related to serine, glycolysis and pentose phosphate metabolism) and three with decreased expression (related to tryptophan and TCA/Krebs cycle metabolism) (Table 5).

**Table 5:** Changes in functionally-defined metabolic pathways in early stage PD.

KEGG Microbial Pathway	Log2 Chg	P value
Tryptophan metabolism (ko00380)	-0.718	0.0081
Formaldehyde assimilation, serine pathway (M00346)	0.315	0.0120
Citrate cycle TCA cycle, Krebs cycle (M00009)	-0.258	0.0385
Citrate cycle TCA cycle (ko00020)	-0.285	0.0457
Glycolysis Embden-Meyerhof pathway, glucose pyruvate (M00001)	0.168	0.0495
Pentose phosphate pathway Pentose phosphate cycle (M00004)	0.418	0.0495

To judge the consistency of microbial metabolic changes in PD subjects, we next used hierarchical clustering of the data within the six altered functional pathways and added whisker-box plots (Fig 3). This indicated relatively consistent separation at the pathway levels. The most visibly shifted pathway based on this analysis was the Tryptophan metabolism pathway (ko00380), which showed a shift of nearly one quartile toward decreased expression in PD.

#### Changes in host salivary transcription networks in early stage PD

In addition to probing for changes in microbial transcripts, taxa, and functional networks, performed similar analyses using host mRNA data from the same subjects. After filtering to identify the most consistently expressed human mRNAs, we tested for group differences using a Student’s t-test and fold change cutoff of +/- 0.5, with FDR correction. This analysis revealed 9 total mRNAs that were significantly different in PD subjects (S6 Table, S3 Fig), and showed a strong ability to distinguish subjects in the two groups (S3 Fig). The biological interactions and ontological enrichments of these 9 genes were examined in STRING. This revealed enrichment in several ontologies considered highly relevant to brain function, including Glial cell proliferation (GO: 0014009), Response to vitamin (GO: 0033273), Response to estradiol (GO:0032355), and Response to oxidative stress (GO:0006979) (S7 Table). The specific genes involved in these processes is displayed in an interaction network (S3 Fig). Two of these nodes contained the same transcripts (Response to vitamin and Response to estradiol) and are shown together.

#### Correlations of differential oral microbiota and host mRNA

To probe potential interactions between the differentially expressed host mRNA and differentially mapped microbiota, we used a Spearman rank correlation analysis, with Bonferroni correction. This revealed a total of 30 significant correlations, all of which were positive, with a maximum Rho value of 0.55 (S8 Table). Notably, of the microbiota, only 2 displayed 4 or more significant correlations (Cellulosimicrobium and Bifidobacterium), and of the human mRNAs, only 3 displayed 5 or more correlations (PENK, EGFLAM and COLGALT1).

#### Correlations of oral microbiome and medical/demographic measures

Our final analysis probed for significant associations among the microbial data and the full set of medical, demographic, and functional outcome measures in an exploratory fashion using Pearson correlation analysis. Because of the large number of correlations generated, we used a conservative approach in interpreting the results of these analyses. The complete set of results are available as supplemental data (S9 Table). Here, we describe only the 10 most robust correlations in magnitude based on absolute rho value (Table 6). Notably, the magnitude of these correlations all exceeded |0.576| with 9 positive associations (all at the species level) and a single negative association (at the genus level). Interestingly, three of the four most robust correlations were with the Duration of PD (years with diagnosis), while the sole negative correlation was with a balance measure (TSEOFP). As already noted, however, this specific functional task did not show significant differences in performance between PD and control groups (Table 3). Among the 10 most robust overall correlations, only one involved a correlation between a significantly changed taxon (*Lactobacillus reuteri*) and a significantly changed functional outcome measure (Trailmaking A time to completion) (Table 6).

**Table 6:** Top microbial correlations with medical/demographic/functional measures.

Taxonomy ID: Genus species	R	Subject Measure
172045: Elizabethkingia miricola	0.645	Duration of PD (years with diagnosis)
526218: Sebaldella termitidis ATCC 33386	0.643	Duration of PD (years with diagnosis)
1112204: Gordonia polyisoprenivorans VH2	0.624	Trailmaking B (time)
1408: Bacillus pumilus	0.618	Duration of PD (years with diagnosis)
1328: Streptococcus anginosus	0.604	Trailmaking B (time)
189423: Streptococcus pneumoniae 670-6B	0.597	Trailmaking B Dual Task (cognitive score)
242231: Neisseria gonorrhoeae FA 1090	0.591	Trailmaking B (time)
1598: Lactobacillus reuteri*	0.588	Trailmaking A (time)
306537: Corynebacterium jeikeium K411	0.576	Trailmaking B (time)
1375: Aerococcus	-0.600	Tandem Stance Eyes Open Foam Pad (sway)

* showed a significant difference (increased abundance) in PD subjects relative to controls

## Discussion

The present study was focused on defining differences in the oral microbiome in early stage PD as determined from shotgun RNA sequencing of saliva samples combined with detailed phenotypic characterization of subjects. We have eight principal findings. First, even in early stage PD, with most subjects on some form of anti-parkinsonian medication, we found evidence of significant (and often highly robust) decreases in balance, sensory, motor and cognitive function. Second, there was no evidence of overall changes in alpha or beta diversity in early stage PD compared with controls. Third, a distinct set of microbial taxa demonstrated consistent changes in sequence abundance at the genus or species level after appropriate correction for multiple testing. Moreover, approximately half of these observed changes fell into clusters of species within the same genera. Fourth, when considered as potential classifiers in a multivariate logistic regression analysis, as few as 11 taxa were found to be capable of distinguishing early stage PD subjects from controls with a 10-fold cross-validated AUC of 0.90 and overall accuracy of 84.5%. Fifth, metabolic pathway analysis of the microbial transcript abundance revealed changes in a distinct subset of biological networks, several of which were highly-related to each other. Sixth, a dual transcriptome analysis revealed strong evidence for changes in abundance of a small set of human mRNAs in PD subjects, many of which are involved in brain or neural functions. Seventh, some of the changes in human mRNAs are significantly correlated with the observed microbial changes. And eighth, exploratory analyses indicated the presence of highly significant correlations between specific microbiota and specific subsets of functional measures, including a robust correlation between one of the significantly changed taxons and one of the changed functional measures. In the space that follows, we briefly discuss the importance of these observations.

### Motor, cognitive, balance and sensory changes in early stage PD

Motor impairments are part of the hallmark symptoms of PD, including bradykinesia and rigidity, and represent two of the criteria used in its diagnosis. Thus, the slowing of reaction times and resulting increase in z scores for the speed-based performance elements that we observed are not surprising and are highly-consistent with a vast literature on the topic. Related to this, it is possible that slowing of movements contributed to reduced performance on the specific cognitive outcome measures that we utilized (SRT, PRT, Trailmaking A and B). Notably, however, approximately half the trials on the GNG task actually involve withholding a response, so this task might be expected to be less affected in its overall score than a purely-motor score if the primary impairment was motor speed alone. However, our data show highly-similar decreases in both SRT and GNG performance (Table 3), suggesting that the decision-making process does exhibit some impairment as well. The possible involvement of reduced motor speed in decreased cognitive task performance is further strengthened by examination of the z score magnitudes for the Trailmaking A and B tasks, since the completion times changed more than the scores themselves compared to controls. Thus, although we clearly cannot separate motor and cognitive performance changes in our PD subject cohort, it appears that increasing cognitive demands of a task results in reduced performance beyond the effects of bradykinesia.

Another hallmark symptom of PD is postural instability. In the motor examination during the UPDRS, this is subjectively evaluated through examination of the patients while standing, walking, turning, and following a pull test. In our cohort of early stage PD subjects, very few individuals exhibited any noticeable impairment in postural stability. Nonetheless, of all the functional measures, the largest z score change in PD subjects was increased body sway compared to controls during a simple static balance task performance (TLEO) (Table 3). This intriguing finding suggests that the computerized functional assessment system we have used to assess PD subjects is highly-sensitive for detecting and quantifying changes in postural sway before they might be obvious or apparent to a trained evaluator.

The final functional domain that we evaluated in our subjects was chemosensory in nature (smell and taste), where our PD cohort scored much worse than the healthy control subjects (Table 3). While not considered pathognomonic, decreased olfaction and taste has been well-documented in PD, including early stages of the disease [26, 27]. Notably, similar decreases in chemosensory function have also been consistently found in subjects with Alzheimer’s disease, or a history of mild traumatic brain injury (mTBI). Thus, our findings are consistent with the literature on early stage PD and suggest that these measures may represent useful screening tools, when used in combination with other assessments, for identifying subjects at risk for neurodegenerative disease in general.

### Comparison of microbiome findings with prior studies

Investigations of the GI microbiome has become increasingly prevalent in the past few years, especially in the case of PD which presents with multiple GI symptoms along with motor symptoms and where pathological changes may be occurring well before CNS involvement [18, 41]. To date, at least a dozen papers have been published on this topic to probe what might be affected in PD. When the results from these 12 studies are compared, several similar changes can be found, even though they were frequently analyzed at varying levels of classification, and most relied on 16S ribosomal RNA gene sequencing for bacterial identification. Specifically, at the family level of classification, despite differences in tissues and fluids tested, there are overlapping findings from many of the studies, though some bacterial families were less consistent. Eleven of the twelve studies analyzed microbiomes in fecal stool samples, while one also compared fecal results to those of sigmoidal colon mucosal biopsies, another compared the fecal results to nasal wash samples, and another investigated potential microbiome changes utilizing oral and nasal swabs [42], although they reported almost no consistent differences. One change that was seen, however, was in the genus Prevotella, which was not changed in our study.

Despite the considerable differences in methodology and tissue sources, the results of the present study are highly-consistent with many of those seen in other studies. In fact, half (8/16) of the bacterial families that we found altered were reported to be altered in prior studies (Table 7). In this report, we focus on two of these families (Bifidobacteriaceae and Lactobacillaceae) which showed similar increases across almost all studies to date and merit further discussion.

**Table 7:** Microbiota families altered in early stage PD in the present study and prior studies.

Microbe Family	Saliva Change	Family Member Changes	Previous Studies	Functional Relevance
Lactobacillaceae	Increase (↑)	Lactobacillus *Lactobacillus acidophilus* *Lactobacillus fermentum* *Lactobacillus plantarum* *Lactobacillus reuteri* *Lactobacillus salivarius* Other Studies *Lactobacillus mucosae* (↑)	Family Pereira^a^^,^^b^ (↑) [42] Hills-Burns ^c^ (↑) [55] Hopfner ^c^ (↑) [56] Scheparjans ^c^ (↑) [51] Bedarf ^c^ (↓) [57] Genus Lactobacillus Hills-Burns ^c^ (↑) [55] Hasegawa ^c^ (↑) [58] Petrov ^c^ (↑) [59] Unger ^c^ (↓) [60] Species/OTU Petrov ^c^ (↑) [59] *L*. *mucosae*	An increase in *L*. *reuteri* lead to greater activity of ENS neurons and vagal afferents, can lead to increased secretion of alpha synuclein [43–45] Increased Lactobacillaceae levels are associated with decreased ghrelin levels [47, 51] Lactobacillus produces GABA and acetylcholine [50] *L*. *reuteri* reduces anxiety and corticosterone in mice [48] Lactobacilli species beneficial in treatment of constipation, diarrhea, and IBS symptoms [49]
Bifidobacteriaceae	Increase (↑)	Bifidobacterium *Bifidobacterium animalis Bifidobacterium dentium Bifidobacterium longum* Gardnerella *Gardnerella vaginalis* Parascardovia *Parascardovia denticolens* Scardovia Other Studies OTU4347159 (Bifidobacterium) (↑)	Family Hills-Burns ^c^ (↑) [55] Bedarf ^c^ (↑) [57] Hopfner ^c^ (↑) [56] Keshavarzian ^d^ (-) [61] Genus Bifidobacterium Unger ^c^ (↑) [60] Hills-Burns ^c^ (↑) [55] Petrov ^c^ (↑) [59] Keshavarzian ^d^ (-) [61] Hasegawa ^c^ (-) [58] Species/OTU Hills-Burns ^c^ (↑) [55] OTU4347159 (Bifidobacterium)	Bifidobacteria have anti-inflammatory properties [11] Bifidobacteria affect local and system immune responses [62] Bifidobacterium produce GABA [50] *B*. *longum* reduces anxious behavior in animals and decreased serum cortisol in humans [63] Bifidobacterium used for treatment for constipation, diarrhea, IBS symptoms, and GI disorders [49] *Gardnerella vaginalis* is associated with bacterial vaginosis [64] *Parascardovia denticolens* is found in dental caries [65]
Saccharomycetaceae	Increase (↑)	Candida *Candida alicans* *Candida duliniensis* *Sacccaromyces cerevisiae* Torulaspora *Torulaspora delbrueckii* Other Studies OTU180999 (Torulaspora) (↓) OTU4325096 (Torulaspora) (↓) OTU4457438 (Torulaspora) (↓) OTU4439469 (Torulaspora) (↑)	Species/OUT Hills-Burns ^c^ (↑) (↓) [55] OTU4439469(Torulaspora) (↑) OTU180999 (Torulaspora) (↓) OTU4325096 (Torulaspora) (↓) OTU4457438 (Torulaspora) (↓)	Suggestive associations of Candida associated oral lesions with increased PD risk in males [66] Candida produces serotonin [50] *Sacccaromyces cerevisiae* produces Ndi1p which can restore function in mice ETC complex 1 that is lost due to Pink1 mutations [53]
Acidaminococcaceae	Increase (↑)	Current and Previous Studies Acidaminococcus	Family Bedarf ^c^ (↑) [57] Genus Acidaminococcus Li ^c^ (↑) [67]	Acidaminococcus consumes glutamate which is important for oxidation in the intestinal epithelium [54]
Vibrionaceae	Increase (↑)	*Lucibacterium_sp_LPB0138*	None	
Brucellaceae	Increase (↑)	Brucella	None	Brucella is the cause of Brucellosis, which can manifest with neurological symptoms, including rare reports of parkinsonism [68–70]
Methylobacteriaceae	Increase (↑)	Methylobacterium	None	
Nocardiaceae	Increase (↑)	Rhodococcus *Rhodococcus_sp_008*	None	*Rhodococcus aurantiacus* induces encephalitis in mice and causes movement disorder mediated by T cell- dependent inflammation; motor symptoms improve with L-DOPA [52]
Microbacteriaceae	Increase (↑)	Clavibacter, *Clavibacter michiganensis*	None	
Promicromonosporaceae	Increase (↑)	Cellulosimicrobium *Cellulomicrobium_sp_TH_20*	None	*Cellulomicrobium sp TH 20* transforms ginsenosides which have anti-inflammatory properties [71]
Enterobacteriaceae	Decrease (↓)	Buchnera, *Buchnera aphidicola*, Other Studies Escherichia (↓)	Family Unger ^c^ (↑) [60] Bedarf ^c^ (↑) [57] Keshavarzian ^d^ (-) [61] Hasegawa ^c^ (-) [58] Genus Escherichia Li ^c^ (↑) [67] Keshavarzian ^d^ (-) [61]	Escherichia produces noradrenaline and serotonin [50] Escherichia is a possible treatment for constipation, IBS, GI disorders, ulcerative colitis, Crohn’s disease, and colon cancer [49] Increase in Enterobacteriaceae is associated with postural instability and gait difficulty phenotype [51]
Rhizobiaceae	Decrease (↓)	*Candidatus azobacteroides Candidatus azobacteroides-pseidotrichonymphae*	None	
Campylobacteraceae	Decrease (↓)	*Campylobacter ureolyticus*	None	Campylobacteraceae implicated in acute GI distress, diarrhea [72]
Streptococceae	Bidirectional (↓) (↑)	*Streptococcus inopinata* (↑) *Streptococcus mutans* (↑) *Streptococcus_phage_PhiSpn 200* (↓) *Streptococcus_sp_I_G2* (↓) Other Studies Streptococcus (↑)	Family Bedarf ^c^ (↓) [57] Genus Streptococcus Li ^c^ (↑) [67]	*S*. *mutans* contributes to tooth decay via production of acidic metabolites [73] Streptococcus produces serotonin [50]
Bacillaceae	Bidirectional (↓) (↑)	*Baccillus megaterium* (↓) *Bacillus_sp_FJAT_2290* (↑) *Halobacillus mangrove* (↓) Other Studies *Incertae sedis XII* (↓)	Family Hopfner ^c^ (↓) [56] Species/OTU Hopfner ^c^ (↓) [56] *Incertae sedis XII*	Bacillus produces noradrenaline and dopamine [50] *Bacillus sp JPJ* produces L-DOPA [74] Bacillus species reduce diarrhea and prevent caries [49]
Flavobacteriaceae	Bidirectional (↓) (↑)	*Capnocytophaga canimorsus* (↑) Chryseobacterium (↓) *Chryseobacterium_sp_IHB_17019* (↓) Wenyingzhuangia (↓) *Wenyingzhuangia fucanilytica* (↓) *Bacterium_3519_10* (↓) Other Studies OTU000509 (Capnocytophaga) (↓) OTU000123 (↓)	Family Pereira^a^^,^^b^ (↓) [42] Bedarf ^c^ (↓)[57] Species/OTU Pereira^a^^,^^b^ (↓) [42] OTU000509 (Capnocytophaga) (↓) OTU000123 (↓)	Flavobacteriaceae have antioxidative properties [75]

Arrows indicate direction of microbiome changes in PD subjects; (-) represents insignificant change; Superscripts indicate tissue source:

^a^, oral;

^b^, nasal;

^c^, fecal;

^d^, colon biopsy

Generally regarded as “probiotic” in nature, bacteria within the Bifidobacteriaceae family are proposed to have anti-inflammatory properties and potentially serve beneficial purposes [11]. Thus, it is possible that the changes we and other groups have seen may reflect a compensatory mechanism in the GI tract. However, while Lactobacilli are also generally considered probiotic, some members of the Lactobacillaceae family may exert a disease-worsening effect in PD. Specifically, *Lactobacillus reuteri*, which we found significantly increased in our PD subjects, was shown in a prior study to increase alpha-synuclein release in cultured ENS neurons, presumably due to increased firing of mesenteric afferent nerve bundles (caused by decreasing calcium-dependent potassium channel opening and reducing the slow afterhyperpolarization in these neurons) [43–45]. In this light, it is particularly worthwhile to note that our exploratory correlation analysis identified a robust positive correlation between the abundance of *Lactobacillus reuteri* and slowing of movement (as reflected in increased performance time on the Trailmaking A test) (Table 6). Other evidence also suggests that Lactobacilli might not be particularly beneficial in PD. Specifically, some members of this bacterial family have been shown to reduce ghrelin secretion, which normally regulates nigrostriatal dopamine and is thought to be neuroprotective, and has been previously reported to be reduced in PD patients [46, 47]. Thus, based on the available data, the consistent increase in Lactobacillaceae we and others have observed in PD may represent a disadvantageous yet consistent event in the disease. This suggestion contrasts with much current opinion regarding Lactobacilli. Indeed, administration of *Lactoballicus reuteri* has been shown to reduce anxiety and corticosterone secretion in mice [48], and several other *Lactobacillus* species have proven beneficial in the treatment of constipation, diarrhea, and IBS symptoms [49] (see Table 7). Accordingly, we suggest that a closer examination of the benefits and risks of *Lactobacillus* supplementation is warranted.

Other findings in our PD cohort are also worth noting because of possible relevance to PD and brain function. Among these include changes in several bacterial families that are known to directly affect neurotransmitter levels. These include *Lactobacillus* and *Bifidobacterium* genus members already discussed, which produce GABA and acetylcholine [50], Enterobacteriaceae family members, which produce norepinephrine and serotonin and are associated with postural instability and gait difficulty phenotypes in PD [51], and members of the Bacillus genus that produce noradrenaline and dopamine [50]. Perhaps the most intriguing finding, however, concerns that of the family Nocardiaceae, which includes the *Rhodococcus* genus and was increased in our early stage PD subjects. The administration of *Rhodococcus aurantiacus* in laboratory mice was shown to induce encephalitis and cause a movement disorder, due to T-cell mediated inflammation, that subsequently responded in a favorable way to L-DOPA treatment [52](Table 7). Thus, the combined set of bacterial families that we observed changed in early stage PD may have broad implications for understanding the pathophysiology of the disorder.

Finally, it is also worthwhile to note that several of the altered microbiota we observed have been linked to PD or are known to play roles in oxidative metabolism. These include members of the Saccharomycetaceae family (encompassing the *Candida* and *Saccaromyces* genera), and members of the Acidaminococcaceae and Flavobacteriaceae families (Table 7). Specifically, *Candida* members produce serotonin and have been anecdotally associated with PD symptoms [50]. In contrast, *Sacccaromyces cerevisiae* produces the rotenone-insensitive NADH:ubiquinone oxidoreductase protein (Ndi1p) which can restore function in complex 1 of the mitochondrial electron transport chain (ETC) that occur due to Pink1 mutations [53]. And *Acidaminococcus* consumes glutamate which is important for oxidation in the intestinal epithelium and is a key contributor to oxidative and amino acid metabolism [54]. These individual taxon findings are further strengthened by the results of our metabolic pathway findings, which highlighted decreases in tryptophan and Krebs cycle metabolism and increases in Glycolysis and Pentose phosphate metabolism in early stage PD. Reductions in Tryptophan metabolism could easily lead to reduced serotonin, melatonin and kyenurenate levels, which have all been shown to have neuroprotective properties. And reduced Krebs cycle metabolism could easily lead to overall decreases in ATP production and increased oxidative stress. Viewed this way, the increased glycolysis and pentose phosphate pathway activities, could therefore represent compensatory attempts to boost ATP production as well as NADPH levels, with a resulting elevation in reduced glutathione levels leading to greater antioxidant capability. Clearly, further studies are needed to test these suggestions and further characterize the metabolomic profiles of the oral microbiome in early stage PD.

Notably, the present study also obtained strong evidence for disruption of a set of human mRNAs in the saliva. Although the origin of these mRNAs was not known, many of these were identified as involved in various neuronal functions (S6 Table), are themselves highly-expressed in the striatum, or map to ontologies which would be considered highly relevant to brain function and PD, such as Glial cell proliferation and Response to oxidative stress (S7 Table). Moreover, we unexpectedly found that several of these same host mRNAs were significantly correlated with differentially expressed microbial taxa (S8 Table). These findings suggest future work on the interactions of host-microbiota may be particularly informative.

## Limitations

There are several limitations worth noting in the present study. First, although the study was moderate in size relative to prior studies in the field, and was focused on early stage PD, it still did not have adequate representation of unmedicated subjects to fully evaluate the consequences of different medications on the outcomes. Second, the results that are described are based on shotgun metatranscriptomic profiling of the oral microbiome which makes direct comparisons with previously published data derived from 16S PCR based approaches somewhat difficult, since 16S reads comprise only a fraction of the available reads for genetic diversity analysis. Third, although the study appears to identify a distinct subset of microbiota that can accurately distinguish early stage PD and control subjects, the true utility of an oral microbiome profile will require establishing the validity of microbiome comparisons between early stage PD subjects and subjects with diagnoses that are commonly confused with or overlap the symptoms of PD, including progressive supranuclear palsy, essential tremor, and multiple system atrophy. Finally, we only quantified the oral microbiome on a single occasion, and it would be very interesting to learn the extent to which dietary and lifestyle factors influence the stability of the outcome. Plans for future studies to address these concerns are currently underway.

## Supporting information

S1 Fig Microbial phylogenetic composition analysis of samples prior to evaluation for group differences. Branch lengths based on a Spearman distance metric.
(TIFF)

S2 Fig Separation of groups according to multidimensional partial least squares discriminant analysis (PLSDA). Only the first 3 principal axes are shown.
(TIF)

S3 Fig Changes in human salivary mRNAs in Parkinson’s subjects. (A), Volcano plot illustrating the fold change compared to the significance of the change in PD subjects, with 9 differential mRNAs identified (red dots). (B), Hierarchical cluster showing the strong separation of PD from control subjects using the 9 differential mRNAs. (C), Gene-gene interaction network with enriched Gene Ontologies superimposed.
(TIF)

S1 Table Raw microbial taxa identified in samples. (XLSX)

S2 Table Raw microbial RNAs identified in samples. RNAs identified according to KO ID.
(XLSX)

S3 Table Raw human mRNAs identified in samples. (XLSX)

S4 Table 40 most abundant microbial RNAs in saliva. RNAs identified according to KO ID.
(XLSX)

S5 Table 40 most differentially abundant microbial RNAs in saliva. (XLSX)

S6 Table Most differential human mRNAs in saliva, and their functions. (XLSX)

S7 Table Most enriched gene ontologies represented by changes human mRNAs. (XLSX)

S8 Table Correlations between changed human mRNAs and microbial taxa. (XLSX)

S9 Table Top correlations of microbiome levels with demographic and clinical features. (XLSX)
