# Supplementary figures and images for: The oral microbiome of early stage Parkinson’s disease and its relationship with functional measures of motor and non-motor function
Source: PLoS One. 2019 Jun 27;14(6):e0218252. doi: 10.1371/journal.pone.0218252 (PMC6597068; doi:10.1371/journal.pone.0218252)

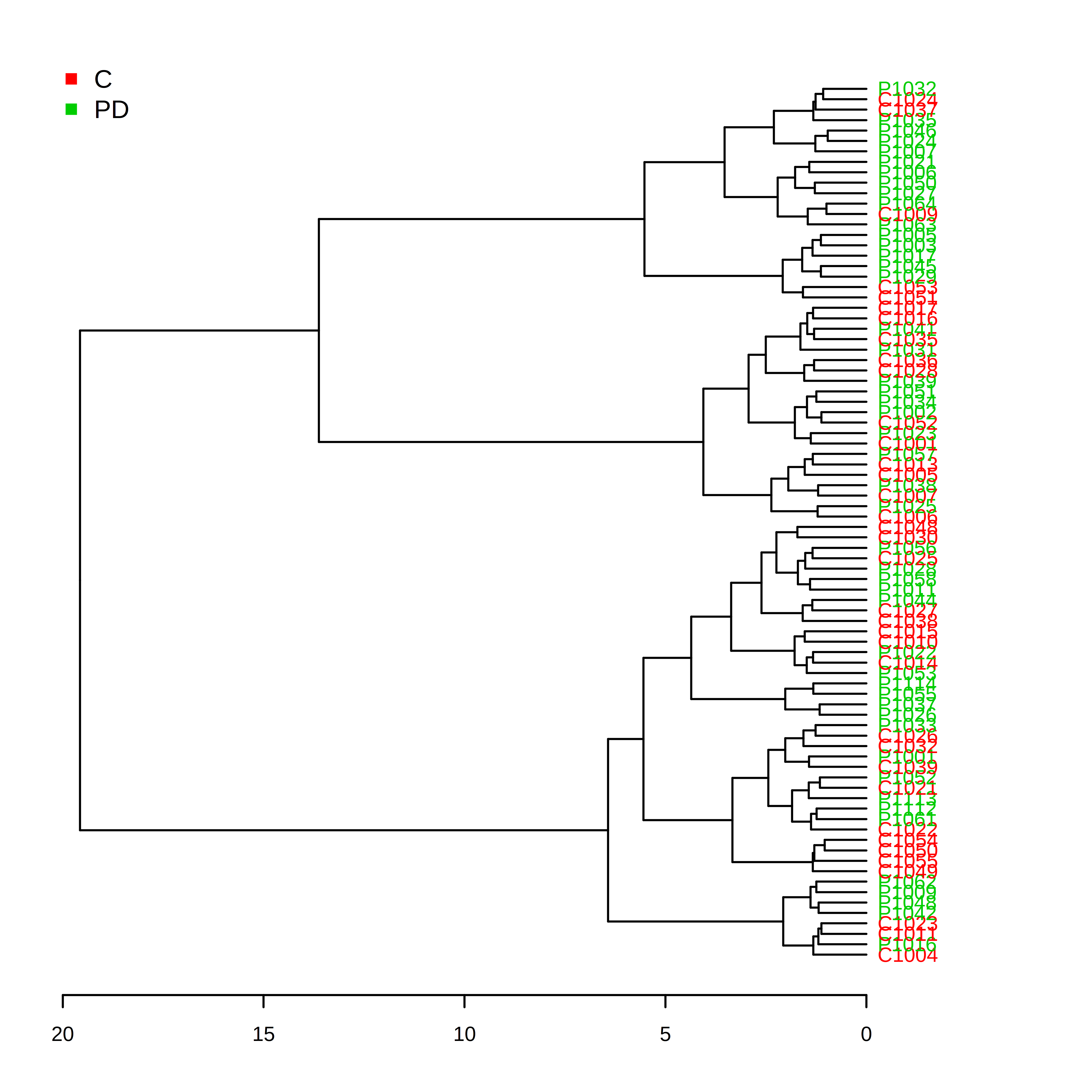

Supplement: S1 Fig — Branch lengths based on a Spearman distance metric. (TIFF) [file pone.0218252.s001.tiff]

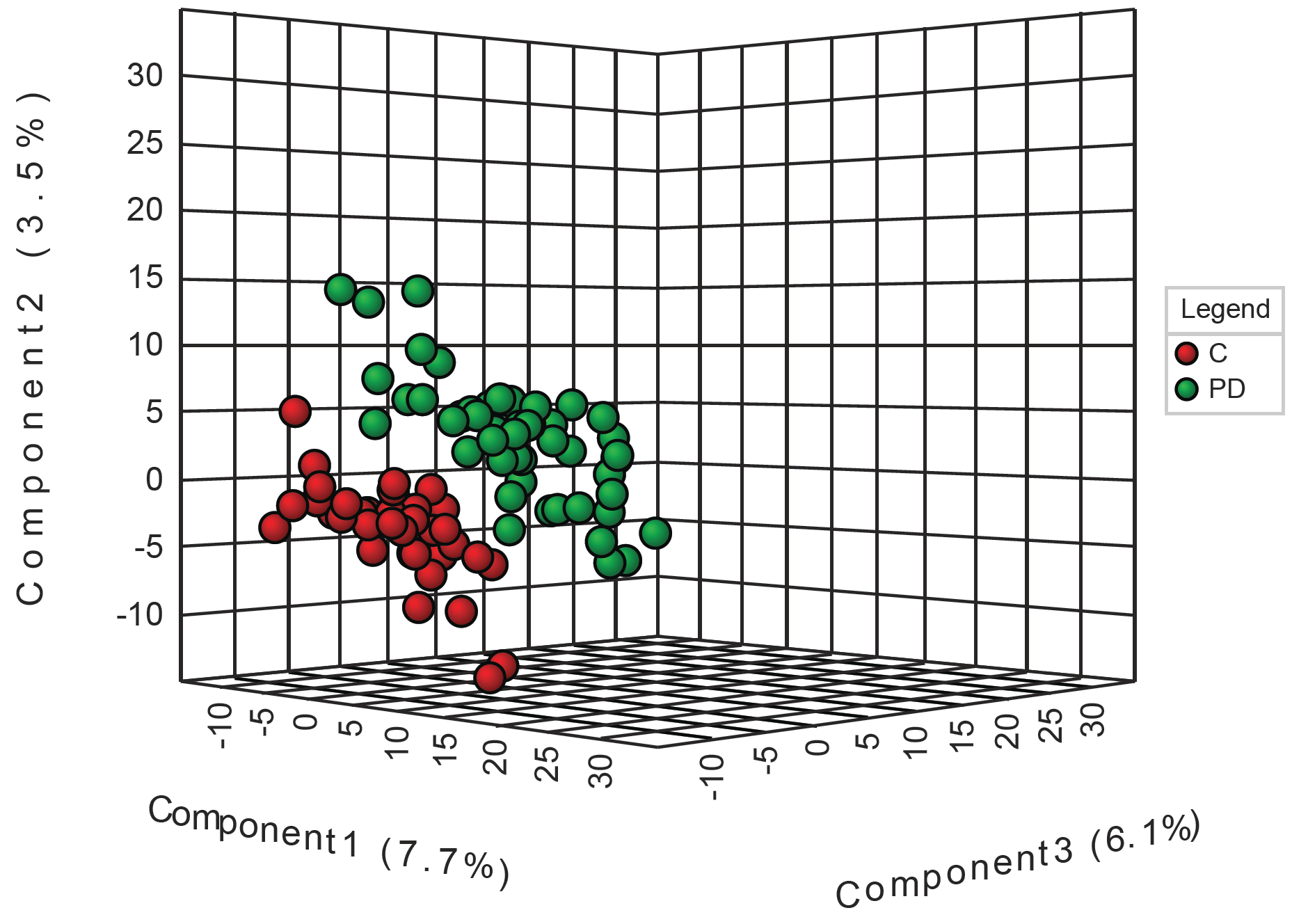

Supplement: S2 Fig — Only the first 3 principal axes are shown. (TIF) [file pone.0218252.s002.tif]

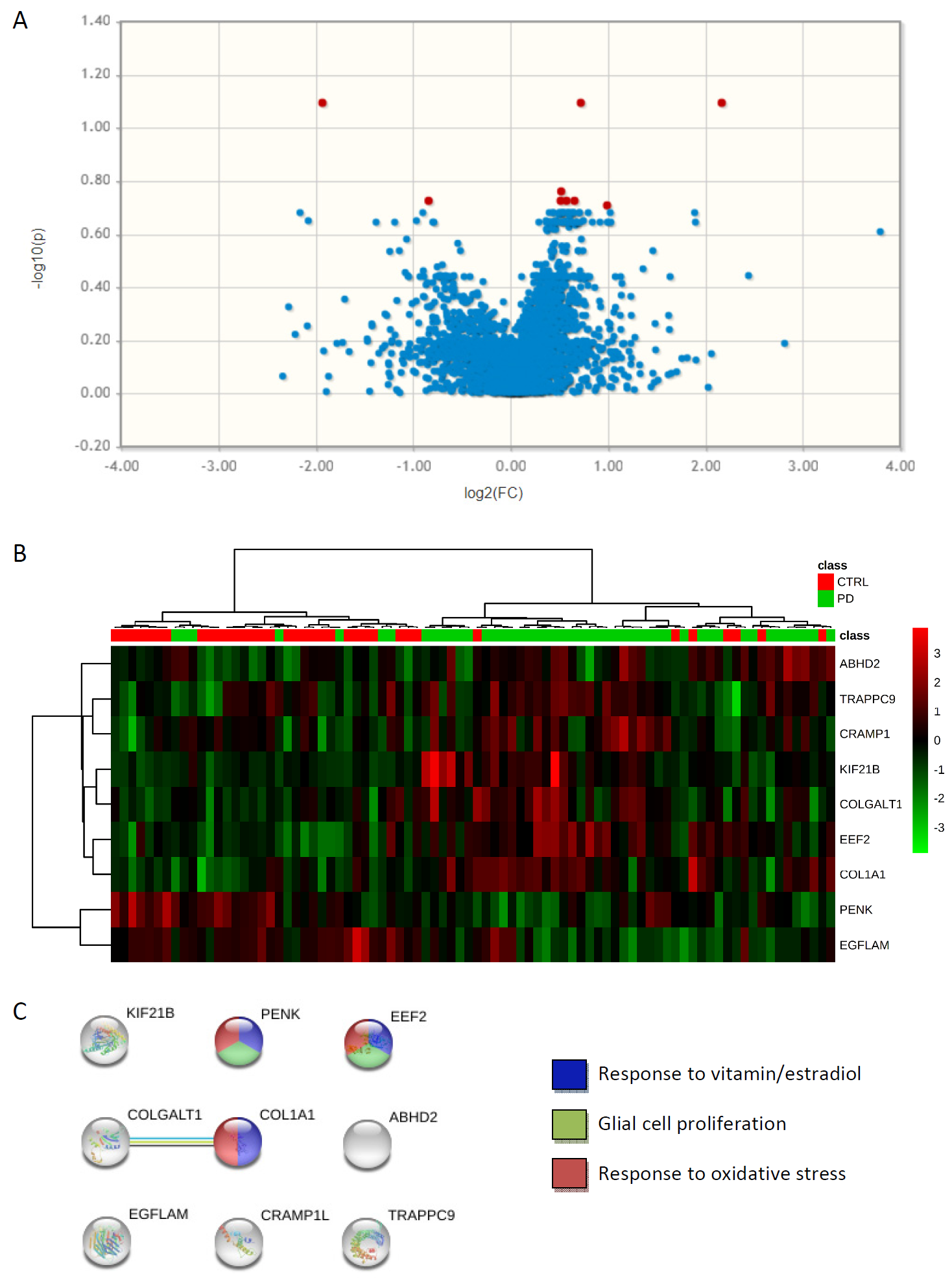

Supplement: S3 Fig — (A), Volcano plot illustrating the fold change compared to the significance of the change in PD subjects, with 9 differential mRNAs identified (red dots). (B), Hierarchical cluster showing the strong separation of PD from control subjects using the 9 differential mRNAs. (C), Gene-gene interaction network with enriched Gene Ontologies superimposed. (TIF) [file pone.0218252.s003.tif]
